# Clinical characteristics of multicentric reticulohistiocytosis and distinguished features from rheumatoid arthritis: a single-center experience in China

**DOI:** 10.1186/s13023-022-02311-y

**Published:** 2022-04-12

**Authors:** Xiao-juan Zou, Lin Qiao, Feng Li, Hua Chen, Yun-jiao Yang, Dong Xu, Wen-Jie Zheng, Zhen-yu Jiang, Li Wang, Qing-jun Wu, Feng-Chun Zhang

**Affiliations:** 1 Department of Rheumatology and Clinical Immunology, Peking Union Medical College, Peking Union Medical College Hospital, Chinese Academy of Medical Sciences, National Clinical Research Center for Dermatologic and Immunologic Diseases, Ministry of Science& Technology, State Key Laboratory of Complex Severe and Rare Diseases, Key Laboratory of Rheumatology and Clinical Immunology, Ministry of Education, Beijing, 100730 China; 2 Department of Rheumatology, First Hospital of Jilin University, Changchun City, Jilin Province China; 3 Department of Dermatology, Peking Union Medical College, Peking Union Medical College Hospital, Chinese Academy of Medical Sciences, Beijing, 100730 China

**Keywords:** Multicentric reticulohistiocytosis, Papulonodular lesions, Erosive polyarthritis

## Abstract

**Objective:**

To investigate the clinical features of multicentric reticulohistiocytosis (MRH).

**Methods:**

The clinical manifestations, laboratory examination results and histologic characteristics of eleven patients with MRH were collected and compared with those of 33 patients with rheumatoid arthritis.

**Results:**

In total, 72.7% of the MRH patients were women. The median age was 46 years (range 33–84 years). Diagnosed by specific pathologic features, all MRH patients exhibited cutaneous involvement. The dorsa of the hands, arms, face and auricle were the most commonly affected areas. Nodules were also located on the legs, scalp, trunk, neck, and even the hypoglossis and buccal mucosa. Ten MRH patients (90.9%) had symmetric polyarthritis. Compared with rheumatoid arthritis (RA) patients, MRH patients were more likely to have distal interphalangeal joint (DIP) involvement (63.6% vs 24.2%, *P* = 0.017) and less likely to have elbow (36.4% vs 72.7%, *P* = 0.003), ankle (45.5% vs 93.9%, *P* < 0.001) and metacarpophalangeal joint (MCP) (36.4% vs 78.8%, *P* = 0.009) involvement. Positivity for rheumatoid factor (RF) (36.4% vs 84.6%, *P* = 0.001) and anti-CCP antibody (9.1% vs 81.8%, *P* = 0.000), as well as the median RF titer [43.8 (31.7–61.0) vs 175.4 (21.3–940.3), *P* = 0.021], in MRH patients was lower than in RA patients. Elevation of the erythrocyte sedimentation rate (ESR) was also less common in MRH patients than in RA patients (36.4% vs 72.7%, *P* = 0.030). After treatment with median- to large-dose corticosteroids and disease-modifying antirheumatic drugs, 8 patients achieved complete remission and 2 patients partial remission (skin lesions ameliorated, joint lesions not ameliorated).

**Conclusion:**

Always pathologically diagnosed, MRH is a systemic disease involving RA-like erosive polyarthritis and a specific distribution of skin nodules characterized by "coral beads". More DIP involvement and less elbow, ankle and MCP involvement are seen in MRH than in RA. In addition, less positivity and lower-titer RF, uncommon presence of anti-CCP antibodies and ESR elevation may be helpful to distinguish MRH from RA.

MRH is a rare rheumatic disease with unclear etiology that is characterized by erosive polyarthritis and typical cutaneous, mucosal or visceral papulonodular lesions, as well as systemic involvement in certain cases. The diagnosis of MRH is always based on biopsy of the nodules, in which multinucleated giant cells and histiocytes with a ground glass appearance of the cytoplasm secondary to lipid inclusions are invariably seen [1]. Because of the nonspecificity of skin manifestations but prominent arthritis in some cases, MRH has at times been misdiagnosed as RA.

As an orphan disease, MRH has only been reported with regard to cases or series, and there is a lack of bench research and clinical trials for better investigation [2]. In this article, the data of 11 MRH cases from our hospital are summarized, and differences from the clinical features of RA are analyzed, by which an improved understanding of this very challenging yet progressive disease is expected.

## Patients and methods

### Patients

Eleven MRH patients admitted to Peking Union Medical College Hospital (PUMCH) from January 2000 to January 2020 were reviewed. All patients were diagnosed with MRH by biopsy and double-verified by at least two dermatologists. Thirty-three (1:3 matched) sex- and age-matched patients with RA were randomized and selected from admitted patients during the same period as controls.

Our study was approved by the Medical Ethics Committee of PUMCH (Beijing, China, approval number: S-K1730). All patients signed written informed consent.

### Statistical analyses

SPSS version 16.0 (SPSS Inc., Chicago, USA) was used to statistically analyze the data. The main results are presented as means and standard deviations (SDs). Significance was estimated by Student’s *t*-test, the χ^2^ test or Fisher’s exact test. *P* values < 0.05 were considered to be statistically significant.

## Results

### Demographic characteristics

Among 11 MRH patients, 8 (72.7%) were women. Their median age was 46 years (range 33–84 years), and their average disease duration was 20 months (range 2–192 months). All patients came from North China.

### Clinical and laboratory characteristics

**Fig. 1:**
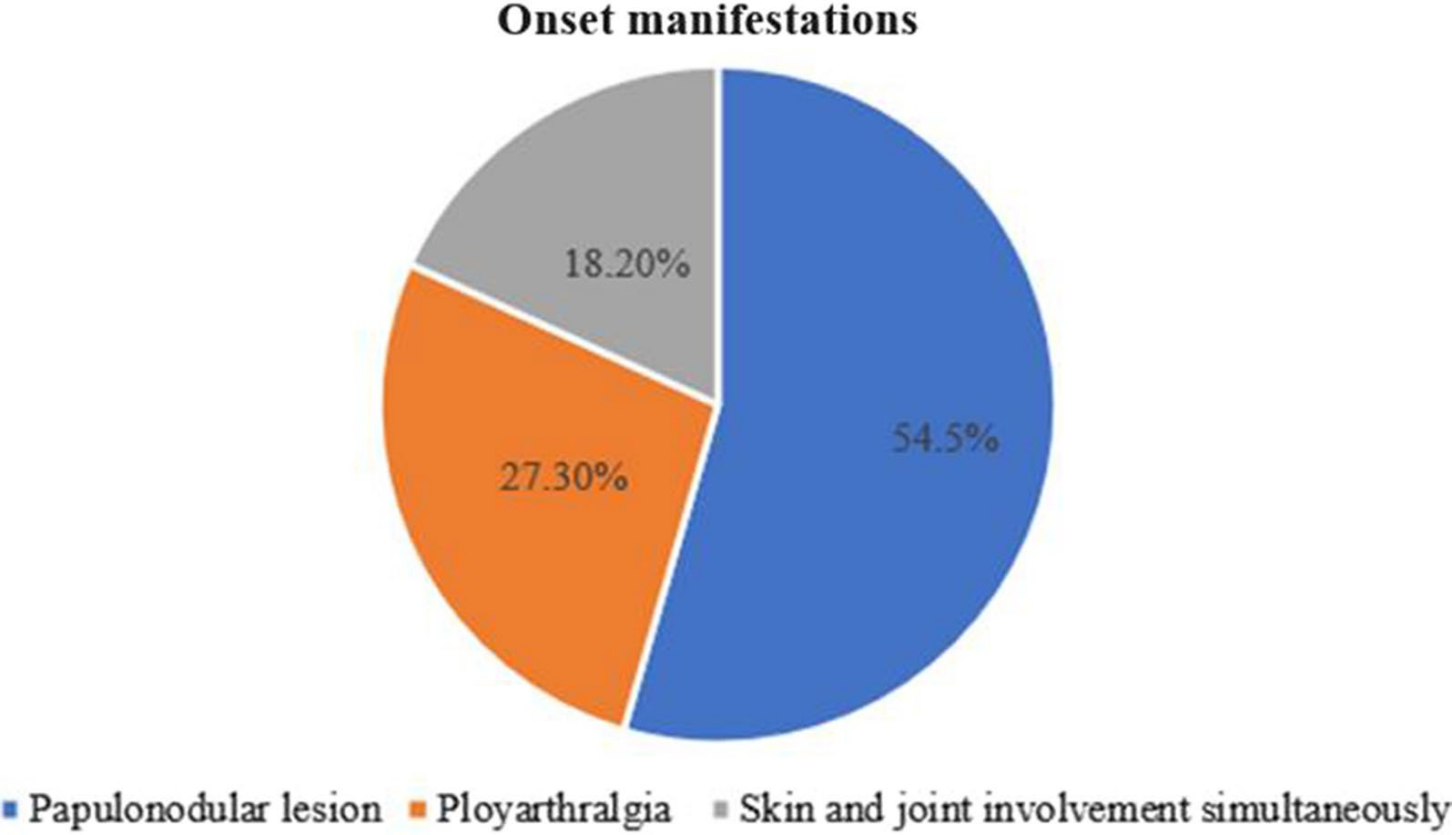
Onset manifestations of MRH patients

Onset manifestations included papulonodular lesions (6/11, 54.5%) and polyarthralgia (3/11, 27.3%). Two patients (2/11, 18.2%) had simultaneous skin and joint involvement (Fig. 1).

### Cutaneous features

**Fig. 2:**
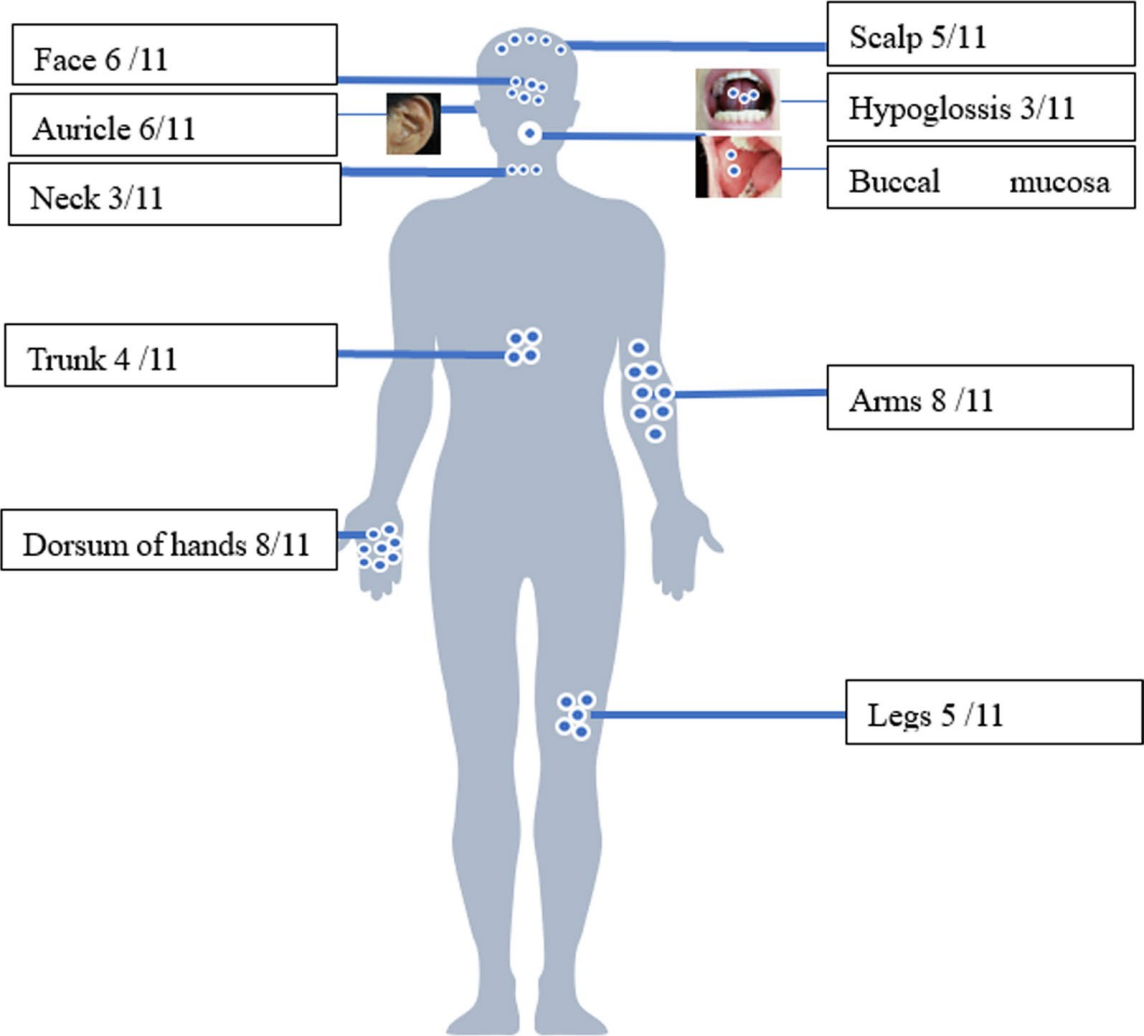
Distribution characteristics of skin nodules in patients with MRH

**Fig. 3:**
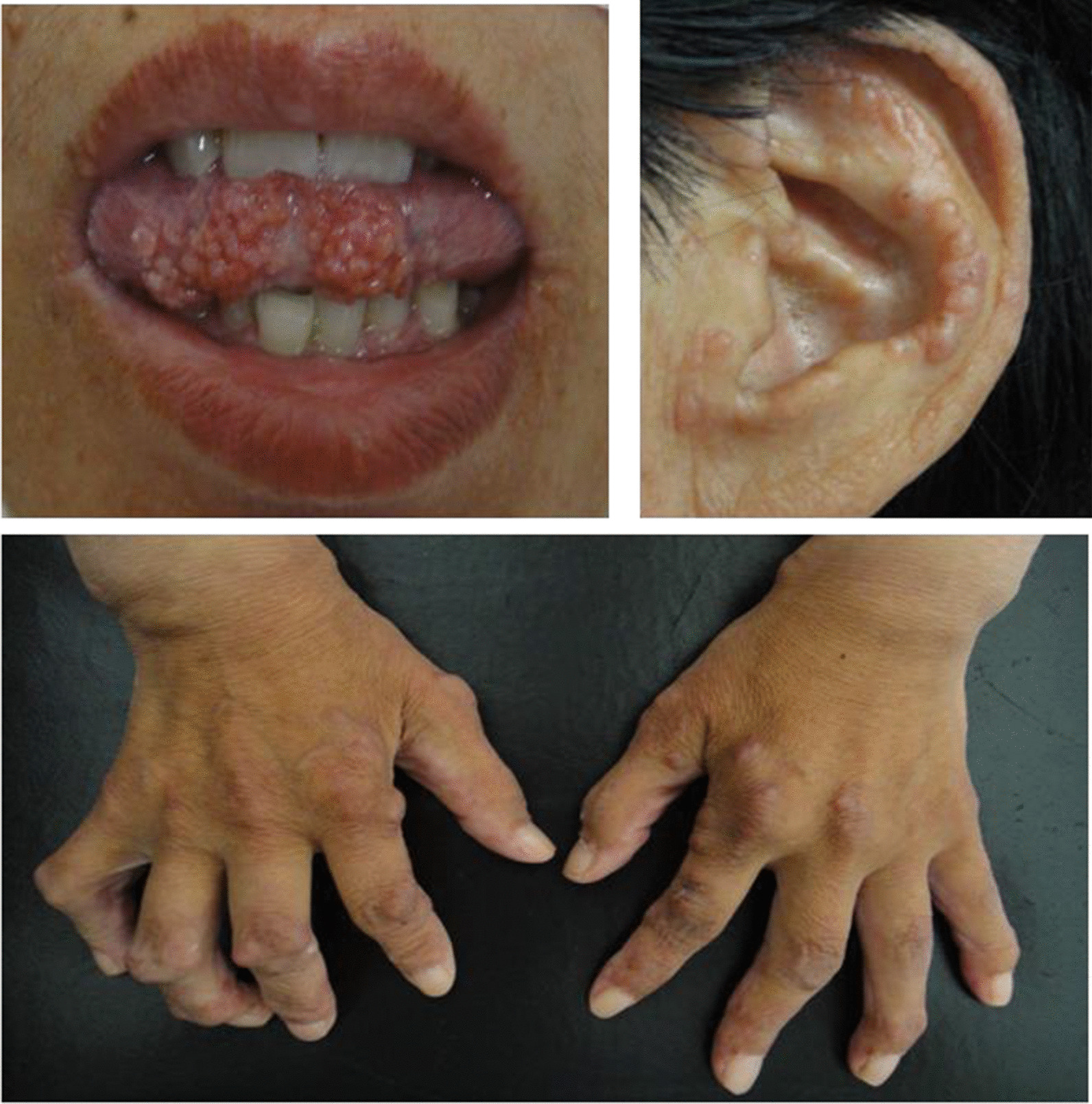
Nodules of the tongue, auricle and dorsum of the hands in MRH patients (from Case 7)

All patients ultimately manifested cutaneous involvement. Brown, purple, reddish or flesh-colored papulonodular lesions occurred in 11 patients (11/11,100%). The dorsa of the hands (8/11, 72.7%, particularly near the knuckles and nail folds), arms (8/11, 72.7%), face (6/11, 54.5%, especially the paranasal areas) and auricle (6/11, 54.5%) were the most commonly affected areas. Nodules were also located on the legs (5/11, 45.5%), scalp (5/11, 45.5%), trunk (4/11, 36.3%), neck (3/11, 27.3%), and even the hypoglossal (3/11, 27.3%) and buccal (2/11, 18.2%) mucosa. Three patients (27.3%) complained of pruritus, and 1 patient (9.1%) experienced tenderness of the nodules (Fig. 2). Most nodules were scattered (7/11); a grouped “cobblestone” appearance occurred in 4 cases (36.3%), while a “coral bead” appearance appeared in 3 cases (27.3%) (Fig. 3).

Diffused-distributed congestive rash was another common skin manifestation (6/11), mostly occurring on the back (5/11), face (4/11), chest (3/11), and neck (2/11).

### Articular features

**Fig. 4:**
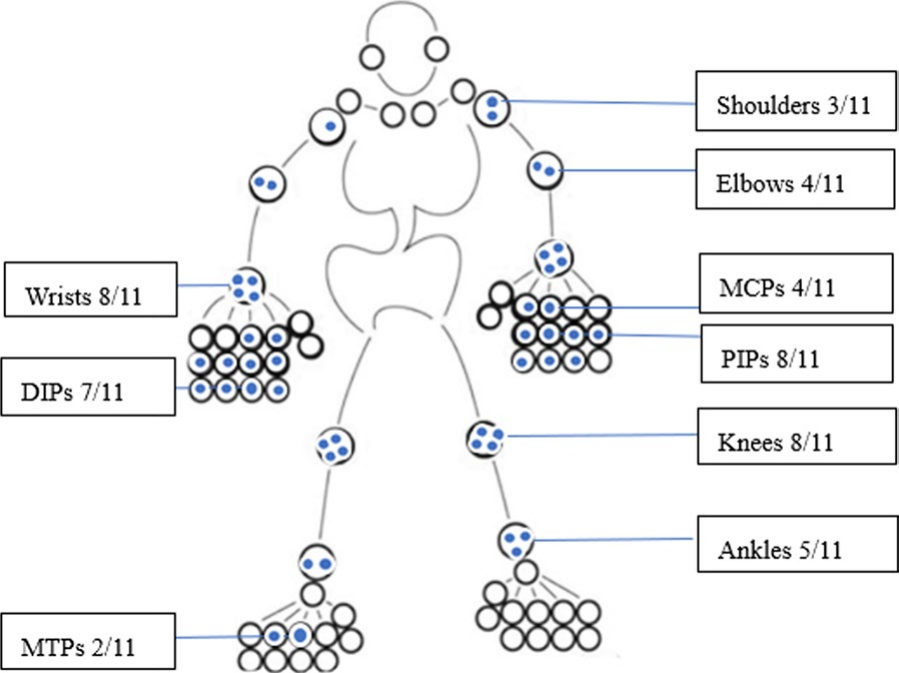
Joint involvement in patients with MRH

Ten patients (90.9%) had symmetric polyarthritis manifested by joint swelling and tenderness, with morning stiffness lasting greater than half an hour; 4 of these patients had deformities. The involved joints included the knees (8/11), proximal interphalangeal joints (PIPs, 8/11), wrists (8/11), distal interphalangeal joint (DIPs, 7/11), ankles (5/11), metacarpophalangeal joints (MCPs, 4/11), elbows (4/11), shoulders (3/11) and metatarsophalangeal joints (MTPs, 2/11) (Fig. 4).

Regarding radiological features, all patients (even those who were symptom free) exhibited destructive changes in the joints. Bone erosion was seen in 7 cases. Four patients exhibited joint-space narrowing, and 6 patients had osteoporosis.

### Systemic involvement and other autoimmune diseases overlapped

Six cases (60%) were associated with the presence of visceral manifestations. Muscular involvement occurred in 4 patients (4/11), manifesting as myalgia and proximal weakness, which were confirmed as myogenic damage by electromyography (EMG). Three patients exhibited multiple pulmonary nodules (3/11), and 1 patient presented fever, fatigue, weight loss, splenomegaly, pleural effusions, and pericarditis (Table 1). Four patients had Sjögren syndrome, while 1 patient had RA.

**Table 1:** Clinical manifestations of MRH patients

No	Age/gender	Duration (months)	Onset symptom	Systemic involvement	Immune markers	Misdiagnosis history	Treatment
1	43/M	12	A	M, L	RF 61.0 IU/ml	RA	Pred 30 mg qd MTX 15 mg qw
2	45/F	20	A	M	ANA(S) 1:20, Ro(SSA) 1:4	SS	Pred 30 mg qd T2 20 mg tid
3	59/M	120	P	M	–	–	Pred 30 mg qd, T 20 mg tid
4	54/F	8	P + A	M	Ro(SSA) 1:4	SS	Pred 30 mg qd, MTX 15 mg qw
5	47/F	72	P	-	ANA(N)1:160	CTD	Pred 50 mg qd, MTX 15 mg qw
6	33/F	24	A	L, S, F	ANA(S)1:80	AOSD	Pred 50 mg qd, CTX 0.4 qw, MTX 10 mg qw
7	47/F	192	A	L	ANA(C)1:80, RF 33.4 IU/ml, Anti-CCP 84 U/ml	RA	Pred 30 mg qd, MTX 12.5 mg qw T 20 mg tid
8	84/M	2	P	–	ANA(H)1:80	–	Pred 30 mg qd, MTX 10 mg qw
9	46/F	12	P + A	–	ANA(S)1:160 Ro(SSA) 1:64 RF 54.2 U/ml	SS	Pred 40 mg qd, CTX 0.4 qw, MTX 10 mg qw
10	46/F	48	P	–	–	–	Pred 50 mg qd, MTX 15 mg qw
11	46/F	15	P	–	ANA:(S)1:80 Ro-52(+ + +), RF: 31.7 IU/ml	CTD	Pred 40 mg qd, MTX 10 mg qw, HCQ 0.2 bid

*Onset symptom* P, Papulonodular lesion; A, Arthritis

*Systemic involvement* M, Muscular involvement; L, Lung disease; S, Splenomegaly; F, Fever

*Immune markers* RF, Rheumatoid factor; ANA; Anti-nuclear antibody; (S), Speckled pattern; (N), Nucleolar pattern; (C), Cytoplasm pattern; (H) Homogenous pattern; Ro(SSA), Anti-Ro(SSA) Antibody; Anti-CCP, Anti- cyclic citrullinated peptide antibody

*Misdiagnosis History* RA, Rheumatoid arthritis; SS, Sjögren syndrome; CTD, Connective tissue disease; AOSD, Adult onset Still disease

*Treatment* Pred, Prednisone; MTX, Methotrexate; CTX, Cyclophosphamide; T, Tripterygium; HCQ, Hydroxychloroquine; qd, once a day; qw, once a week; tid, 3 times a day; bid, 2 times a day

### Laboratory findings

There were no abnormal changes in routine examinations of blood or urine or in biochemical markers of liver and kidney functions in patients with MRH. Elevated erythrocyte sedimentation rates (ESRs) and C-reactive protein (CRP) levels were seen in 4 patients (4/11). Rheumatoid factor (RF) was detected (positive) in 4 patients (4/11), and the median titer was 43.8 (31.7–61.0) IU/ml. Seven patients exhibited positivity for antinuclear antibodies, 4 of whom had anti-SSA antibodies (Table 1).

### Comparison with RA

Compared with RA patients, MRH patients were more likely to have DIP involvement (63.6% vs 24.2%, *P* = 0.017) and less likely to have elbow (36.4% vs 72.7%, *P* = 0.003), ankle (45.5% vs 93.9%, *P* < 0.001) and MCP (36.4% vs 78.8%, *P* = 0.009) joint involvement, which were statistically significant.

**Fig. 5:**
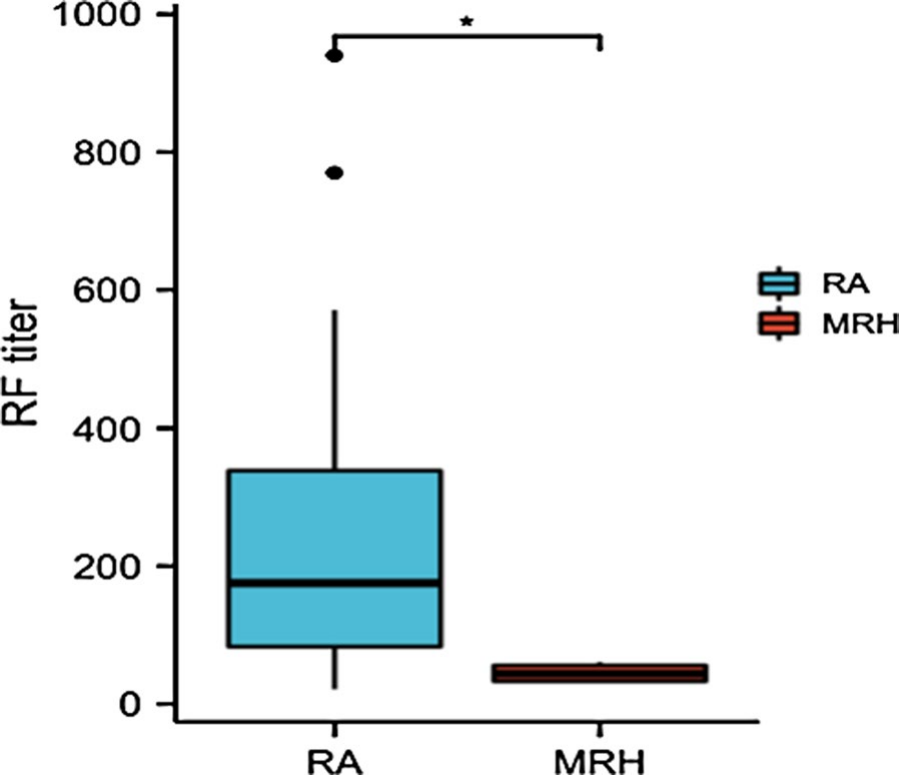
Comparison of the RF titer in RF-positive patients between RA and MRH

Positivity for RF (36.4% vs 84.6%, *P* = 0.001) and anti-CCP antibody (9.1% vs 81.8%, *P* = 0.000), as well as the median RF titer [43.8 (31.7–61.0) vs 175.4 (21.3–940.3), *P* = 0.021] (Fig. 5), in MRH patients was lower than that in RA patients. Elevation of ESR was less common in MRH patients than in RA patients (36.4% vs 72.7%, *P* = 0.030) and showed significance. There was no significant difference in age, sex, antinuclear antibody (ANA), C-reactive protein (CRP) or other joint involvement, including knee joints, PIP joints, wrist joints and shoulder joints, between patients with MRH and those with RA (Table 2).

**Table 2:** Clinical comparison of MRH with RA patients

Characteristics	MRH (N = 11)	RA(N = 33)	*P* value
Age (years)	46 (33–84)	54(25–69)	0.416
Gender (F/M)	8/3	29/4	0.234
PIPs involved (n, %)	8 (72.7)	31 (93.9)	0.055
Knees involved (n, %)	8 (72.7)	29 (87.9)	0.234
Wrists involved (n, %)	8 (72.7)	31 (93.9)	0.055
DIPs involved (n, %)	7 (63.6)	8 (24.2)	0.017*
Ankles involved (n, %)	5 (45.5)	32 (97.0)	< 0.001*
MCPs involved (n, %)	4 (36.4)	26 (78.8)	0.009*
Elbows involved (n, %)	4 (36.4)	24 (72.7)	0.030*
Shoulders involved (n, %)	3 (27.3)	15 (45.5)	0.288
Elevated CRP (n, %)	4 (36.4)	19 (57.6)	0.223
Abnormal ESR (n, %)	4 (36.4)	24 (72.7)	0.030*
Positive RF (n, %)	4 (36.4)	29 (87.9)	0.001*
RF titer (IU/ml, median, range)	43.8 (31.7–61.0)	175.4 (21.3–940.3)	0.021*
Positive anti-CCP (n, %)	1 (9.1)	27 (81.8)	0.000*
Positive ANA (n, %)	7 (63.6)	16 (48.5)	0.384

PIP, Proximal interphalangeal joint; DIP, Distal interphalangeal joint; MCP, Metatarsophalangeal joint; CRP, C-reactive protein; ESR, Erythrocyte sedimentation rate; RF, Rheumatoid factor; CCP, Cyclic citrullinated peptide antibody; ANA, Antinuclear antibody

*P values < 0.05 were considered to be statistically significant

### Histopathology

All patients underwent biopsy of skin nodules. The lesions were characterized by multinucleated giant cells with a ground glass eosinophilic cytoplasm. Additional staining included positivity for CD68 (11/11, 100%), CD45 (2/11, 18.2%), and CD43 (1/11, 9.1%).

In addition, several patients underwent biopsies of the synovium (No. 5), lung (No. 1) and bronchial mucosa (No. 6). Similar pathologic changes were seen in these tissues as those from skin lesions in 4 patients, and muscle biopsies did not show any changes.

### Treatments and prognosis

All cases were treated with glucocorticoids (GCs) combined with immuno suppressants. The mean onset dosage of prednisone was 37.3 (30–50) mg/d. methotrexate (9/11) and Tripterygium (3/11) were commonly selected as the immunosuppressants. In addition, 2 patients were treated with cyclophosphamide (one menopausal patient and one critically ill patient with multiple organ system involvement who did not respond well to other medications). Hydroxychloroquine was added for 1 patient.

All patients improved and were discharged. During follow-up ranging from 6 months to 2 years, 8 patients achieved complete remission (resolution of both skin and joint symptoms), 2 patients achieved partial remission (skin lesions improved, but joint symptoms remained), and 1 patient relapsed after steroid tapering. No patients died.

## Discussion

MRH is an orphan multisystem inflammatory disease with an unknown etiology. MRH predominantly occurs between the ages of 40 and 50, but it can also be diagnosed in infants, children and elderly individuals; women are more commonly affected than men, at a ratio of 3:1 [3]. Our research also suggested that women might have a higher incidence (8/11). MRH is characterized by mutilating arthritis and multiple reticular histiocytic nodules in the skin and mucosa. Most of the patients have outstanding skin manifestations, but in others, joint symptoms may be more dominant. RA can also show multijoint lesions and rheumatoid nodules, which is the reason MRH is sometimes misdiagnosed as RA. In our study, MRH patients with onset manifestations that included papulonodular lesions and polyarthralgia also exhibited simultaneous skin and joint involvement. In 3/11 cases involving articular onset, the patients might have been easily misdiagnosed with RA. In 6/11 patients, the disease began with skin signs and symptoms, and such cases might have been referred to a dermatologist. Arthritis associated with MRH tends to be symmetrical and erosive in a polyarticular pattern including both large and small joints that mimic RA, but its erosive change seems to be more important than synovitis. In our study, almost all the patients (10/11, 90.9%) experienced symmetric polyarthritis that was more inclined to the knees (8/11), PIPs (8/11), wrists (8/11), DIPs (7/11) and ankles (5/11). Although all synovium-rich joints can be affected in RA, the MCPs, PIPs and wrists are the most typical sites. According to the comparison of these two diseases in this study, a higher prevalence of DIP involvement seemed to be one characteristic manifestation distinguishing MRH from RA. At the same time, elbow, ankle and MCP involvement may be relatively less common in MRH than in RA, which is consistent with most previous reports [4]. Sanchez-Alvarez et al. [5] reported that typical hand X-ray in MRH patients showed symmetrical destructive polyarthritis, accompanied by joint subluxation, angular deformity and articular surface erosion, which mainly affected the DIPs, but had fewer erosive changes in the PIPs, MCPs and wrists. One limitation of this study was its lack of an imaging comparison between the two diseases, which needs further investigation.

In our research, skin lesions of MRH were largely characterized by "coral beads" of skin nodules around the nail and could also appear explosively and symmetrically on the arms, face, torso and mucosal surfaces. Rheumatoid nodules, the most common cutaneous manifestation of RA [6, 7], are usually seen on pressure points and are separately distributed (such as on the olecranon process) and found in internal organs, such as the lungs [8]. The diagnostic method of MRH mainly depends on the typical pathological changes, which include the presence of histiocytes and multinucleated giant cells with ground glass cytoplasm. The cells are eosinophilic, homogeneous or fine grained, with a ground glass appearance, and periodic acid–Schiff PAS staining results are positive. Immunohistochemical staining shows positivity for CD68 [9]. The typical histologic feature of rheumatoid nodules is necrobiosis, with fibrin deposition and palisading epithelioid histiocytes [10–12]. When the patients' symptoms are atypical, they need to be pathologically differentiated.

Twenty percent of MRH patients have lung involvement, and their cases are sometimes complicated with myositis or serositis [13]. In our study, more than half of the patients had organ involvement, among which myalgia or proximal muscle weakness and pulmonary nodules were common, much higher than in previous reports. The reason might be more complicated cases for admitted patients than outpatients. As a comparison, extra-articular involvement (e.g., vasculitis, scleritis, interstitial lung diseases) occurs in approximately 40% of RA patients over a lifetime [14, 15]. Varilla et al. believed that MRH patients may not only be diagnosed with connective tissue diseases (CTDs), such as RA, lupus erythematosus, Sjögren syndrome and dermatomyositis [16, 17], but may also have cases overlapping with these CTDs. In our study, positive ANAs and anti-SSA antibodies were observed in patients with MRH, which also may suggest that there may be some relationship between MRH and CTDs.

In addition, RF and anti-CCP antibodies have diagnostic meaning in RA but are rare in patients with MRH. In our study, there were significant differences in positivity of RF and anti-CCP antibodies between RA and MRH (*P* < 0.05). Although positive, the titers of the RF or anti-CCP antibody were not very high, and the four patients’ indicators did not meet RA classification criteria. The titer of RF in the MRH group was significantly lower than that in the RA group. ESR elevation is regularly seen in RA patients but rare in MRH patients. This might suggest that inflammation is less severe in MRH. Kumar et al. analyzed synovial fluid from a portion of the studied patients and found that it was noninflammatory [18], which means that RA involves synovitis, while MRH seems to result in direct joint-bone destruction with less inflammation.

There are no guidelines or expert recommendations on how to treat MRH. Corticosteroids are not necessary as a first-line choice in the treatment of RA or as a bridging therapy at low doses and in the short term. In our study, most MRH patients were treated with a median dose as a first-line agent. The reasons might include arthritis that is more destructive than RA, dermatitis that is often quite disfiguring [19] and a higher incidence of extra-articular involvement in MRH [20]. As far as steroid-sparing agents are concerned, the choice of methotrexate and Tripterygium (which has been included as a DMARD in China's Clinical diagnosis and treatment Guidelines for RA [21–24]) might be references for the treatment of RA. The initial price of the biologics was relatively high and medical insurance in China could not cover them during these years, which might be the most important reason why these patients did not choose these drugs. However, biologics might be a beneficial choice for MRH patients [5, 18, 25–27]. In fact, it seemed that most patients with MRH had a satisfactory response to these medications. Nevertheless, several limitations existed in the present study. There were two orphan diseases involving MRH, and the statistical results were exploratory. As a retrospective study, its analysis was descriptive and preliminary and lacked further investigation of the pathogenesis and prognosis. In addition, we did not perform joint-imaging data analysis and comparison, which is also worthy of additional analysis.

## Conclusion

In conclusion, MRH is a rare disease with RA-like erosive polyarthritis. In addition to showing the typical explosive and symmetrical skin nodules characterized by "coral beads", MRH patients were more likely to have DIP involvement and less MCP, elbow and ankle joint involvement. In addition, less positive and lower-titer RF, uncommon anti-CCP antibody and elevation of ESR may be helpful in distinguishing MRH from RA.

## Data Availability

The datasets generated and/or analyzed during the current study are not publicly available but are available from the corresponding author on reasonable request.
